# Axillary Aneurism

**Published:** 1829-04

**Authors:** 


### AXILLARY ANEURISM.

*Case of Axillary Aneurism, at the* PHILADELPHIA ALMSHOUSE. By Professor GIBSON.

THIS is a highly interesting and instructive case. The patient, a stout, muscular, athletic man, about six feet high, applied to Professor GIBSON on account of a luxation of the left os humeri at the shoulder-joint, of nine weeks' standing. He was admitted into the Almshouse Infirmary on the 6th of March: the antiphlogistic system was pursued until the 15th, when attempts at reduction were made, in the presence of the surgeons and students of the house, which was not accomplished until after the lapse of an hour and three-quarters from the commencement of the operation.

On the 16th, there was a general swelling over the deltoid and pectoral muscles, with a distinct pulsation of an aneurismal character.

On the morning of the 17th it had increased considerably, and in consultation "it was decided that the subclavian artery should be tied without delay." This was accordingly done by Professor Gibson.

A note is here appended, descriptive of an instrument invented at his suggestion, for passing a ligature around deep-seated arteries, to which we can bear testimony as being probably the best that has yet been devised for that purpose. "It consists of a silver canula, fixed on a wooden handle, surrounded (near the part where the canula joins the handle) with a collar, through which a steel stilet, made of a narrow watchspring, the length of the instrument, passes, and immediately afterwards enters an opening just below the collar, in order to traverse the whole cavity of the canula, and emerge at its point. This extremity of the stilet is covered with a flattened silver cap moderately blunt; whilst its other, or upper extremity, passing upwards from the collar above mentioned, lays parallel with the handle, and has an eye near its end for holding a ligature. A small screw, for the purpose of fixing the stilet while the surgeon is in the act of passing the instrument beneath the artery, works through the silver collar, and may be used or not, as the surgeon pleases."

Our readers will hardly consider it necessary for us to enter into the minute details of the progress of the case, which are carefully recorded. We shall briefly state, therefore, that the patient died at four o'clock P.M. on the 25th, the tenth day after the ligature of the subclavian.

The account of this case concludes with the dissection, which was conducted by Dr. ASHMEAD, (the parts having been previously injected,) and the report drawn up by Dr. HALL, in the presence of a large number of physicians and students. It is so highly interesting, and important for a full understanding of the case, that we conceive it matter of justice to Dr. Gibson, and requiring no apology to give it entire.

" *Philadelphia Almshouse; March 24th,* 1828.

" *Dissection of John Langton, sixteen hours after death.*—The left hand and forearm exhibited marks of incipient gangrene, extending only to the skin and subjacent cellular membrane, and terminated by a well-defined line at the elbow. The wound made by the operation was filled with an offensive sanies, and exhibited no tendency to healthy granulations.

" We threw in the cold lead injection, by fixing a pipe in the mouth of the left subclavian, through the aorta: the shoulder and neck were minutely injected, but, as the radial artery was not filled, the pipe was introduced into it, and the lead driven upwards until a portion of the first injection was made to recede through the divided thoracic vessels.

" The arm, scapula, and clavicle, with a portion of the ribs, were separated from the body, and carefully dissected by Dr. Ashmead and myself, in the presence of the surgeons and house students. All the parts about the axilla were so blended by adhesions as to render their discrimination very difficult. The muscles were unaltered, except from extravasation and effusion to be hereafter described. The subclavian artery was healthy, the vertebral of its usual caliber; the internal mammary large; the inferior thyroideal, the posterior cervical, and the superior scapular, arose by a common trunk from the subclavian, together with an anomalous artery, about the size of a crowquill, which descended between the subclavian and carotid, along the trachea; its destination was not pursued. The posterior cervical was of the size of a large quill, having its branch to the base of the scapula much enlarged. The superior scapular artery ran along the upper edge of the subclavius muscle, and had escaped the operator's knife, though it had been made visible by the incisions. It inosculated beneath the neck of the scapula with the inferior dorsalis scapulæ, which was of the size of a crowquill. From the unfavorable state of the part for dissection, we could not demonstrate the channels through which the blood to the arm passed; but that these existed, and would, under auspicuous circumstances, have become adequate to the circulation, the course of the injection and other circumstances fully prove.

" As the injection from above had penetrated to the ligature, it is presumed that no coagulum had as yet formed. The ligature embraced the artery just where it emerges from beneath the scalenus anticus, and included no other part. The artery was uninjured in all the space between the ligature and the point where it became adherent to the head of the bone, at the internal margin of the lesser tubercle. It was firmly attached to the substance of the bone and the articular capsule by dense cellular or ligamentous substance; *and such was the compactness of this juncture, and so short the portion of artery between this point and that of its attachment to the rib, that it seemed absolutely impossible to reduce the bone to its place and not cause the rupture of the vessel.* This effect was here exhibited. Where the artery adhered to the bone, its internal coats had been ruptured, with the exception of a very narrow band immediately opposite to its point of attachment. The extremities of the artery had separated, as far as the band above alluded to would allow, (which was about half an inch,) and the artery, by tending to straighten itself, was only retained to the bone by the intervening portion of its external coat. The dilatation of the external coat had formed a sac, which was expanded by being stretched between the points of its attachment to the bone and the ruptured extremity of the artery. This true aneurismal sac extended beneath the artery towards the ligature, and beneath the pectoralis minor muscle, but was ruptured in its posterior portion, very near its adhesion to the bone, so as to have allowed the blood to escape and form a diffused aneurism. The blood had penetrated beneath the pectoralis major and minor muscles, and along the edge of the latissimus dorsi as far as the seventh rib. It had extended beneath the humerus to the space between the long head of the triceps and the teres minor, and had filled the axillary cavity, but had been prevented from extending downwards by the general agglutination of the parts, caused by the dislocation. Along the internal margin of the coraco brachialis and the deltoid, there was much extravasation; but we rather think it was caused by the means used to reduce the bone.

" The walls of the true aneurismal sac were of so compact a texture, and its boundaries so well defined, that the conjecture of its having existed previously to the reduction of the bone, and that its rupture was a distant and subsequent event, is rendered probable.

" Upon exposing the articular cavity, the head of the bone was found to rest beneath its original socket upon a bed of dense ligamentous substance. The capsule was much thickened, and had a rupture in its inferior anterior portion, through which the blood and injecting matter had entered.

" About one third of the lower portion of the glenoid cavity had been broken off, and remained attached to the superior part of the neck of the bone, by an adventitious adhesion.

" The greater tubercle of the humerus was cracked through its base, with the exception of the portion beneath its anterior facet. From the thickening of the periosteum, and the deposit of osseous matter, we do not think that this fracture was recent. The extremity of the acromion process was found fractured, but was still firmly embraced by the surrounding muscular and fibrous structure.

" It is proper to mention that the left ventricle of the heart was enlarged, and its muscular substance unusually soft. The right ventricle and auricles did not exhibit these phenomena. Upon opening that part of the subclavian embraced by the ligature, the internal coats of the vessel were found completely divided, but no traces of a coagulum could be observed above the ligature; nor was there any vestige of coagulable lymph, or any approach to adhesion, though the ligature held its place with great tenacity, and the parts embraced by it seemed healthy.

" No other part was examined.

" James C. Hall, M.D."

Some remarks, growing out of the unfortunate results of this, which is the second case of the kind that has occurred to Professor Gibson, and the only two cases that have been reported in this country, follow. Allusion is first made to those who have advised, and successfully executed, the reduction of old luxations of the humerus. There can be no doubt of the fact that the practice is warranted by experience; and, for ourselves, we should not hesitate a moment to undertake it, if a fair case were presented to our notice. The cases of Foubert, (as well as several others,) which were alluded to in our last Number, page 451, are next adduced, to show that the same results have followed similar attempts by other surgeons; and from these Dr. G. has drawn some valuable practical inferences, to which we shall call attention at the conclusion of this article.

" It is not my intention," says Dr. G. " to comment, except in a very brief way, upon the case of Langton. The particulars of it having been fairly and honestly stated, the profession will be able to draw its own conclusions. I may remark, however, that between the first accident of the kind I have detailed and the second, (a period of five years,) I have reduced five luxations, each from two to four months' standing: one a patient of Dr. D. T. Coxe, upwards of sixty years old; another of Dr. Manuel E. Robinson, about the same age; and three more in my own practice; besides several similar accidents of a recent kind, all of which terminated in the happiest manner.

"From these, and from many other cases of a similar character which I have treated within the last eighteen years, I think I have good reason to conclude that, where no adhesion exists between the artery and surrounding parts, the operation may be done with safety: that, on the contrary, when adhesion does exist, (and of this we have no means to judge,) rupture of the vessel must be an inevitable consequence, whether the reduction be effected by force or by the most gentle means. To show, however, that neither my colleagues nor myself made use of unwarrantable force in the case of Langton, I shall present the following documents, obligingly furnished by some of the gentlemen present at the reduction."*

The concluding observations of Dr. G. respecting the reduction of old luxations, are practically valuable, and we shall conclude by submitting them to our readers.

"If the patient is young, not very muscular, the luxation not complicated with fracture; if no attempts have previously been made to accomplish the reduction, and the head of the bone has not been out of its natural situation beyond five or six weeks, I should advise the attempt to replace it. But, on the contrary, if the patient is very robust and vigorous, advanced in years, accustomed to labour and to the free use of ardent spirits, and the head of the bone has been long out, I should discountenance any attempt at reduction."—*American Medical Recorder.*

* The documents referred to confirm the statements of Dr. G.—EDITORS.

